# Comment on: Non-invasive measurement of intestinal tissue oxygen saturation for evaluation of reconstructed blood flow in rectal cancer surgery: HiSCO-09 study

**DOI:** 10.1093/bjs/znad399

**Published:** 2023-12-07

**Authors:** Iannish D Sadien

**Affiliations:** Cancer Research UK Cambridge Institute, University of Cambridge, Li Ka Shing Centre, Cambridge CB2 0RE, UK

*Dear Editor*

Yoshinaka and colleagues^1^ presented an interesting study evaluating tissue oxygen saturation (r*S*o_2_) measured by near-infrared spectroscopy as a tool to assess intestinal blood flow during colorectal surgery. They found that r*S*o_2_ was higher after preservation of the left colic artery (LCA), and that low r*S*o_2_ at the anastomotic site was an independent risk factor for anastomotic leak. Although these are intriguing findings, several limitations should be considered.

First, this was an observational study without randomization between arterial preservation and resection. Propensity score matching (PSM) was used to adjust for confounders, but residual bias likely remains. An RCT would provide higher-quality evidence for the benefit of LCA preservation.

Second, the authors noted that, along with r*S*o_2_, Eastern Cooperative Oncology Group performance status, longer operating time, LCA resection, and lower tumour location were associated with anastomotic leak in multivariable analysis. However, the study design does not allow determination of whether low r*S*o_2_ causes anastomotic leak or is simply a marker of complex surgery. This could have been addressed using PSM, for example, comparing high *versus* low r*S*o_2_.

r*S*o_2_ depends on the rate of blood flow through the tissue coupled with the oxygen saturation of that blood. It would therefore have been judicious to include perioperative blood oxygen saturation as a variable in the regression.

Finally, with the data provided, it is unclear what the sensitivity and specificity of the 74% r*S*o_2_ cut-off was for predicting anastomotic leak. It is unknown whether this could meaningfully change clinical practice, as many leaks could still occur above this threshold.

In summary, although measuring tissue oxygenation is biologically plausible for assessing intestinal perfusion, the real-world impact of this specific device requires further investigation. Carefully designed randomized trials are required to determine its role in colorectal surgery.
